# GOALS OF CARE IN END-STAGE RENAL DISEASE: THE INFLUENCE OF AGE AND MULTIMORBIDITY

**DOI:** 10.1093/geroni/igz038.450

**Published:** 2019-11-08

**Authors:** Deborah P Waldrop, Patricia Denny, Sandra Lauer, Kathleen Grimm, Phyllis Murawski, Mandip Panesar

**Affiliations:** 1 University at Buffalo School of Social Work, Buffalo, New York, United States; 2 Erie County Medical Center, Buffalo, New York, United States

## Abstract

The number of people with End Stage Renal Disease (ESRD) who need dialysis treatment has increased sharply among adults age 75+. Older adults on dialysis have lower rates of advance care planning and higher treatment intensity, hospitalization and intensive care than people other chronic illnesses. Comprehensive care of older adults with ESRD includes advance care planning that addresses goals of care and not just specific medical treatments. The purpose of this study was to explore the nature of symptom burden and advance care planning in dialysis patients. The study design was exploratory, descriptive and cross-sectional. Quantitative and qualitative data were collected during in-person chairside interviews with people having dialysis treatments. Categorical questions focused on demographics and advance directives. The Dialysis Symptom Inventory was used to measure symptom burden. Open-ended questions addressed the trajectory of illness and goals of care. Thirty-five interviews were conducted. Participants’ Mage=55.8 years (range 27-84); 51 % were >60. A distinctive pattern of difference by age emerged. Participants >60 demonstrated greater multimorbidity and lower symptom burden (MDSI=30.13; Range 11-63) compared with those <60 (MDSI=36.31; Range 3-78). Goals of care also varied with age. Older adults’ goals were: (1) Functional (e.g. to walk better, drive); and (2) Existential (e.g. maintaining, surviving, enjoying). Goals of participants <60 were: (1) Transplantation; and (2) Engagement (e.g. work, school, travel). The results suggest that the illness experience and goals are influenced by age and multimorbidity. Implications: ESRD-specific advance care planning conversations with a focus on goals of care are important.

